# Virological Failure and Mortality Among People Living with HIV Transitioned to Second- or Third-Line Antiretroviral Therapy in Rwanda: A Competing-Risks Survival Analysis of National Case-Based Surveillance Data, 2019–2025

**DOI:** 10.3390/v18080905

**Published:** 2026-08-17

**Authors:** Gallican N. Rwibasira, Jean Claude Kwizera, Tafadzwa Dzinamarira, Steven Karera, Gatete Gaetan, Albert Tuyishime, Eric Remera, Daniel Henry Paris, Tracy R. Glass

**Affiliations:** 1 Rwanda Biomedical Centre, Kigali P.O. Box 7162, Rwanda; claude.kwizera@rbc.gov.rw (J.C.K.); gaetan.gatete@rbc.gov.rw (G.G.); albert.tuyishime@rbc.gov.rw (A.T.); eric.remera@rbc.gov.rw (E.R.); 2 Swiss Tropical and Public Health Institute, 4002 Basel, Switzerland; daniel.paris@swisstph.ch (D.H.P.); tracy.glass@swisstph.ch (T.R.G.); 3 Faculty of Medicine, University of Basel, 4056 Basel, Switzerland; 4 ICAP Global Health, Rwanda Country Office, Kigali P.O. Box 1524, Rwanda; td2581@cumc.columbia.edu; 5 ICAP Global Health, Zambia Country Office, Lusaka P.O. Box 34358, Zambia; sk5391@cumc.columbia.edu

**Keywords:** second-line antiretroviral therapy, virological failure, competing risks, case-based surveillance, Rwanda

## Abstract

Background: Despite Rwanda’s achievement of the UNAIDS 95–95–95 targets, national evidence on virological failure (VF) and mortality after transition to second- or third-line antiretroviral therapy (ART) remains limited. We estimated the incidence of these outcomes and examined associated factors among people living with HIV (PLWH) transitioned to second- or third-line ART in Rwanda between 2019 and 2025. Methods: We conducted a retrospective cohort study using Rwanda’s national HIV case-based surveillance (CBS) system. PLWH aged ≥15 years who transitioned to second- or third-line ART between 1 January 2019 and 31 December 2025 were followed from transition until the first recorded outcome, last clinical contact, or administrative censoring. The primary operational VF endpoint was the first viral load >1000 copies/mL recorded ≥180 days after transition; a prespecified sensitivity analysis required two consecutive measurements >1000 copies/mL. We used Kaplan–Meier estimation and facility-clustered Cox regression for the composite outcome of VF or death, and Aalen–Johansen cumulative incidence functions and Fine–Gray regression for VF with death treated as a competing event. Results: Among 778 PLWH followed for 3564 person-years (median follow-up, 60.0 months), 81 (10.4%) met the primary operational VF endpoint, and 13 (1.7%) had death recorded as the first event. The composite incidence rate was 2.64 per 100 person-years (95% CI 2.10–3.17). In adjusted Cox regression, a PI-based regimen (adjusted hazard ratio [aHR] 2.14, 95% CI 1.37–3.36), WHO stage III (aHR 1.92, 95% CI 1.10–3.34), and WHO stage IV (aHR 3.59, 95% CI 1.61–8.02) were associated with the composite outcome. In Fine–Gray analysis, a PI-based regimen (subdistribution hazard ratio [sHR] 3.37, 95% CI 1.96–5.78) and WHO stage IV (sHR 4.44, 95% CI 1.91–10.4) were associated with VF. Conclusions: PI-based regimen use and advanced WHO clinical stage were associated with poorer outcomes after ART line transition. These findings support intensified viral load monitoring, adherence and resistance assessment, and individualized regimen review for patients receiving PI-based therapy, together with systematic implementation of the WHO advanced HIV disease package for patients with stage III or IV disease.

## 1. Background

Globally, there were an estimated 40.8 million people living with HIV (PLWH) in 2024, with sub-Saharan Africa (SSA) remaining the epicentre of the epidemic. SSA is home to at least 26.3 million PLWH and accounts for half of all people who acquired HIV globally in 2024, having nonetheless achieved a 56% decline in new infections since 2010 [1]. The large-scale rollout of antiretroviral therapy (ART) beginning in the mid-2000s has transformed the trajectory of the epidemic: since the start of the epidemic, an estimated 26.9 million deaths have been averted through treatment [1,2]. In 2024, 630,000 people died from AIDS-related causes, 61% of them in SSA—a profound reduction from peak mortality, yet a reminder that the epidemic remains far from over [1].

Despite these achievements, virological non-suppression remains an important programmatic challenge. WHO guidance classifies a viral load >1000 copies/mL as unsuppressed and generally defines virological failure as persistent non-suppression confirmed by a second measurement after adherence support in a person who has received ART for at least six months [3]. A 2025 systematic review estimated a pooled prevalence of virological failure of 19.4% (95% CI 15.2–24.0%) in East Africa [4]. Reported estimates vary substantially by ART line, outcome definition, monitoring intensity, and study setting [5,6,7,8,9].

The introduction of dolutegravir (DTG)-based regimens has improved virological outcomes and offers a higher genetic barrier to resistance than earlier regimens [10,11,12,13]. Nevertheless, some patients require transition to second- or third-line therapy [3]. These regimens are more complex to manage, may require resistance-informed selection, and are less well characterized in national programme data across sub-Saharan Africa [9,14].

Rwanda has achieved the UNAIDS 95–95–95 treatment targets: among an estimated 230,000 PLWH, 96% know their status, 98% of those diagnosed receive treatment, and 98% of those receiving treatment are virally suppressed [1]. Rwanda’s HIV case-based surveillance (CBS) system, implemented on the DHIS2 platform, links individual-level enrolment, laboratory, treatment, and follow-up records from HIV service-delivery facilities [15,16]. This longitudinal platform provides an opportunity to examine programme outcomes after transition to second- or third-line ART at the national level.

Advanced WHO clinical stage, low CD4 cell count, tuberculosis co-infection, poor adherence, delayed regimen transition, and high viral load at transition have been associated with poor outcomes on second-line ART [9,14,17]. Social factors may also influence adherence and retention [8,17,18], but their role after transition to second- or third-line ART has not been examined nationally in Rwanda. When VF and death are analysed as mutually exclusive first events, death precludes subsequent observation of VF and should therefore be handled as a competing event when estimating the cumulative incidence of VF [19].

This study aimed to (i) estimate the incidence of the primary operational VF endpoint and recorded all-cause death among PLWH transitioned to second- or third-line ART in Rwanda between 2019 and 2025; (ii) identify demographic and clinical factors associated with the composite outcome of VF or death; and (iii) estimate the cumulative incidence of VF and its associated factors while treating death as a competing event.

## 2. Methods

### 2.1. Study Design and Setting

We conducted a retrospective cohort study using Rwanda’s national HIV case-based surveillance (CBS) system. Rwanda is a landlocked country in East–Central Africa with a population of approximately 14 million [20]. The CBS links enrolment data from CRF1 (demographic, clinical, laboratory, and initial treatment information) with longitudinal CRF2 records (viral load results, WHO clinical stage, regimen, adherence assessment, and programme outcome) across HIV service-delivery facilities coordinated by the Rwanda Biomedical Centre [15].

### 2.2. Study Population

We included PLWH aged ≥15 years with a documented transition to a second- or third-line ART regimen between 1 January 2019 and 31 December 2025. Eligible transitions were identified from CRF1 records documenting a regimen change and from CRF2 follow-up records documenting a new second- or third-line regimen; the recorded ART change date was used as the transition date. Patients with a missing transition date or unique patient identifier were excluded.

### 2.3. Outcome Definitions

The primary composite outcome was the time from ART transition to the first occurrence of the operational VF endpoint or recorded all-cause death. The operational VF endpoint was the first viral load (VL) >1000 copies/mL recorded at least 180 days after transition; because VF cannot be ascertained before this window, VF-specific analyses (cumulative incidence and Fine–Gray regression) used delayed entry, with the risk set beginning at day 180 post-transition, while death and the composite outcome remained observable from day 0. Because WHO-defined virological failure generally requires confirmation after adherence support [3], a prespecified sensitivity analysis required two consecutive VL measurements >1000 copies/mL after the six-month window (Figure S1, Table S1). Death was defined from the recorded CRF2 programme outcome and dated using the documented outcome date. For competing-risk analyses, VF and death were treated as mutually exclusive first events.

### 2.4. Follow-Up and Censoring

Patients were followed from the ART transition date until the earliest of the operational VF endpoint, recorded death, last clinical contact, or administrative censoring on 31 December 2025. Patients recorded as stopped were classified as lost to follow-up and censored at their last recorded contact. Follow-up for the composite outcome began at transition, although VF was only ascertainable from day 180 onward. Censoring was assumed to be non-informative in the primary analysis; because loss to follow-up in routine care may be associated with unrecorded outcomes, a sensitivity analysis re-estimated the composite incidence rate under extreme-value assumptions in which 10% or 25% of censored patients were assumed to have experienced an unrecorded event (Table S2).

### 2.5. Exposure Variables

The covariates included sex; age at transition, summarized descriptively in five bands (<25, 25–34, 35–44, 45–54, or ≥55 years) and, to preserve events-per-parameter in the regression models, grouped into three bands (<35, 35–54, or ≥55 years) for adjustment; WHO clinical stage (I–IV); CD4 count at transition (<200, 200–499, ≥500 cells/µL, or unknown); and regimen class at transition: integrase strand transfer inhibitor (INSTI)-based, protease inhibitor (PI)-based, or other. Marital and employment status were taken from the CRF2 record nearest the transition date, preferring a record on or before transition date and using the nearest subsequent record only if none was available beforehand, and were treated as fixed covariates. Adherence was summarized descriptively as the worst clinician-recorded category after transition; because it was post-baseline and missing for most patients, it was not included in the adjusted models. Adherence was recorded as a categorical clinician assessment (good, moderate, or bad) per Rwanda’s national ART programme M&E tools; CBS does not capture the specific counselling criteria (e.g., pill count or missed-dose recall) underlying this categorical judgement.

### 2.6. Statistical Analysis

Continuous variables were summarized using medians and interquartile ranges, and categorical variables using frequencies and percentages. Kaplan–Meier methods and log-rank tests described event-free survival for the composite outcome. Cox proportional hazards models estimated unadjusted and adjusted hazard ratios, with robust standard errors clustered by health facility; the proportional hazards assumption was assessed using Schoenfeld residuals. Adjusted models included sex, age group (3-level), WHO clinical stage, CD4 category, regimen class, marital status, and employment status. For VF, cumulative incidence functions were estimated using the Aalen–Johansen estimator, and Fine–Gray regression, with delayed entry from day 180 and robust standard errors clustered by health facility, treated death as the competing event. A death-specific Fine–Gray model was not fitted because only 13 deaths occurred as first events. Given the modest number of events relative to model parameters, events-per-parameter was calculated for both the composite Cox model and the VF-specific Fine–Gray model. The adjusted Cox model was refit using the sensitivity VF definition (Table S3). Analyses were conducted in R statistical software, version 4.5.1 (R Foundation for Statistical Computing, Vienna, Austria, 2025), using the survival (version 3.8.3), gtsummary (version 2.4.0), ggsurvfit (version 1.2.0), and tidycmprsk (version 1.1.2) packages. Two-sided *p* < 0.05 was considered statistically significant.

### 2.7. Ethics

This study used de-identified data collected through Rwanda’s routine HIV case-based surveillance system. The CBS programme was launched jointly by the Rwanda Biomedical Centre and the US Centers for Disease Control and Prevention, approved in 2018 (tracking number 2018-309), and granted a non-research determination in 2021. The surveillance protocol provides for verbal informed consent; adolescents aged 15–17 years may consent under an approved waiver of parental permission.

Only de-identified programme data were used for this analysis, and no personal identifiers were shared with the study team or third parties.

## 3. Results

### 3.1. Cohort Characteristics

A total of 778 PLWH met the eligibility criteria. The median age at ART transition was 44 years (IQR 33–51), and 519 (67%) were female. At transition, 517 (66%) were in WHO stage I, 126 (16%) in stage III, and 25 (3.2%) in stage IV. The median CD4 count was 391 cells/µL (IQR 247–591). Most participants transitioned to second-line ART (757, 97%); 21 (2.7%) transitioned to third-line ART. Regimens were INSTI-based for 428 (55%) and PI-based for 350 (45%). Adherence was documented for 123 patients (16%); because 84% had no adherence record, these data should be interpreted cautiously. Baseline characteristics are shown in Table 1.

### 3.2. Events and Follow-Up

Over 3564 person-years of follow-up (median 60.0 months), 81 patients (10.4%) met the operational VF endpoint before a recorded death, and 13 (1.7%) had death recorded as the first event. The composite outcome occurred in 94 patients (12.1%), corresponding to 2.64 events per 100 person-years (95% CI 2.10–3.17). The VF incidence rate was 2.27 per 100 person-years (95% CI 1.78–2.77), and the reported mortality rate was 0.36 per 100 person-years (95% CI 0.17–0.56). The remaining 684 patients (87.9%) were censored. Table 2 summarizes first events and incidence rates.

### 3.3. Kaplan–Meier Survival Analysis

**Figure 1:**
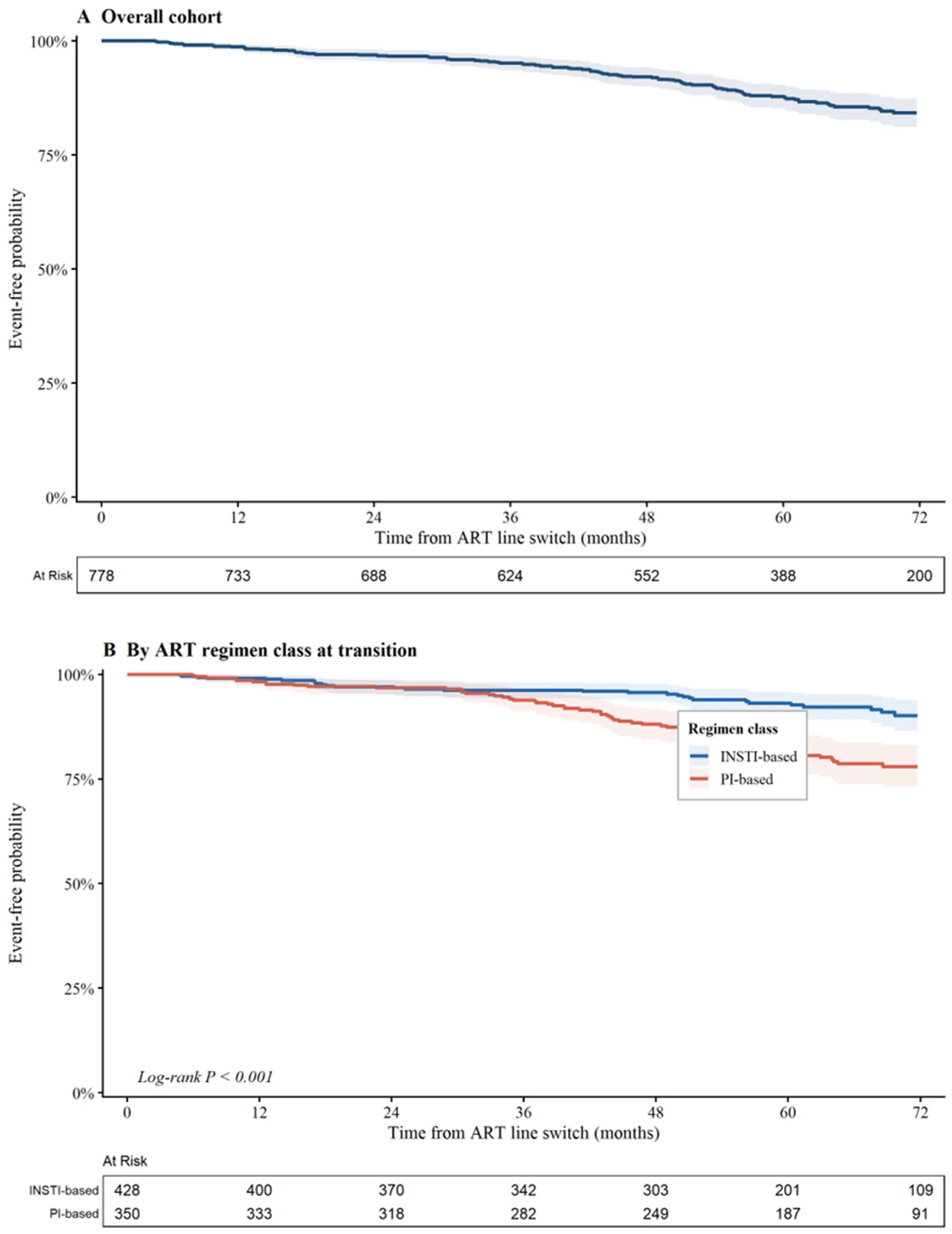
Kaplan–Meier event-free survival curves for time to the composite outcome (virologic failure or death) among PLWH transitioned to second- or third-line ART, Rwanda CBS cohort, 2019–2025. (**A**): Overall cohort. (**B**): Stratified by ART regimen class after line transition (other excluded). Shaded bands = 95% confidence intervals. Numbers at risk are displayed below each panel. Log-rank *p*-value shown for stratified comparison.

**Figure 2:**
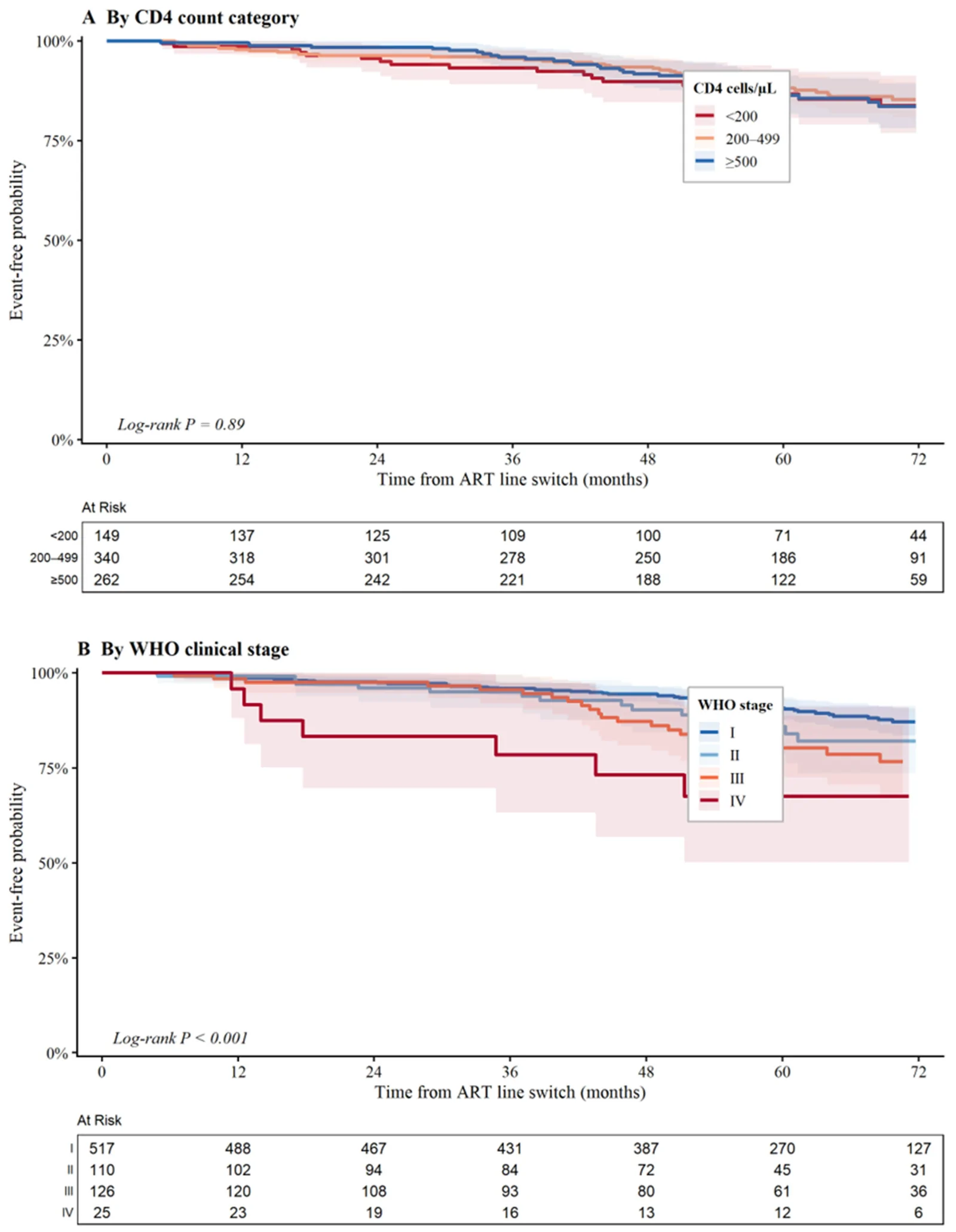
Kaplan–Meier event-free survival curves for time to the composite outcome (virologic failure or death), stratified by baseline CD4 count category (**A**) and WHO clinical stage (**B**). Rwanda CBS cohort, 2019–2025. Shaded bands = 95% confidence intervals. Numbers at risk are displayed below each panel. Log-rank *p*-values shown for each comparison.

**Figure 3:**
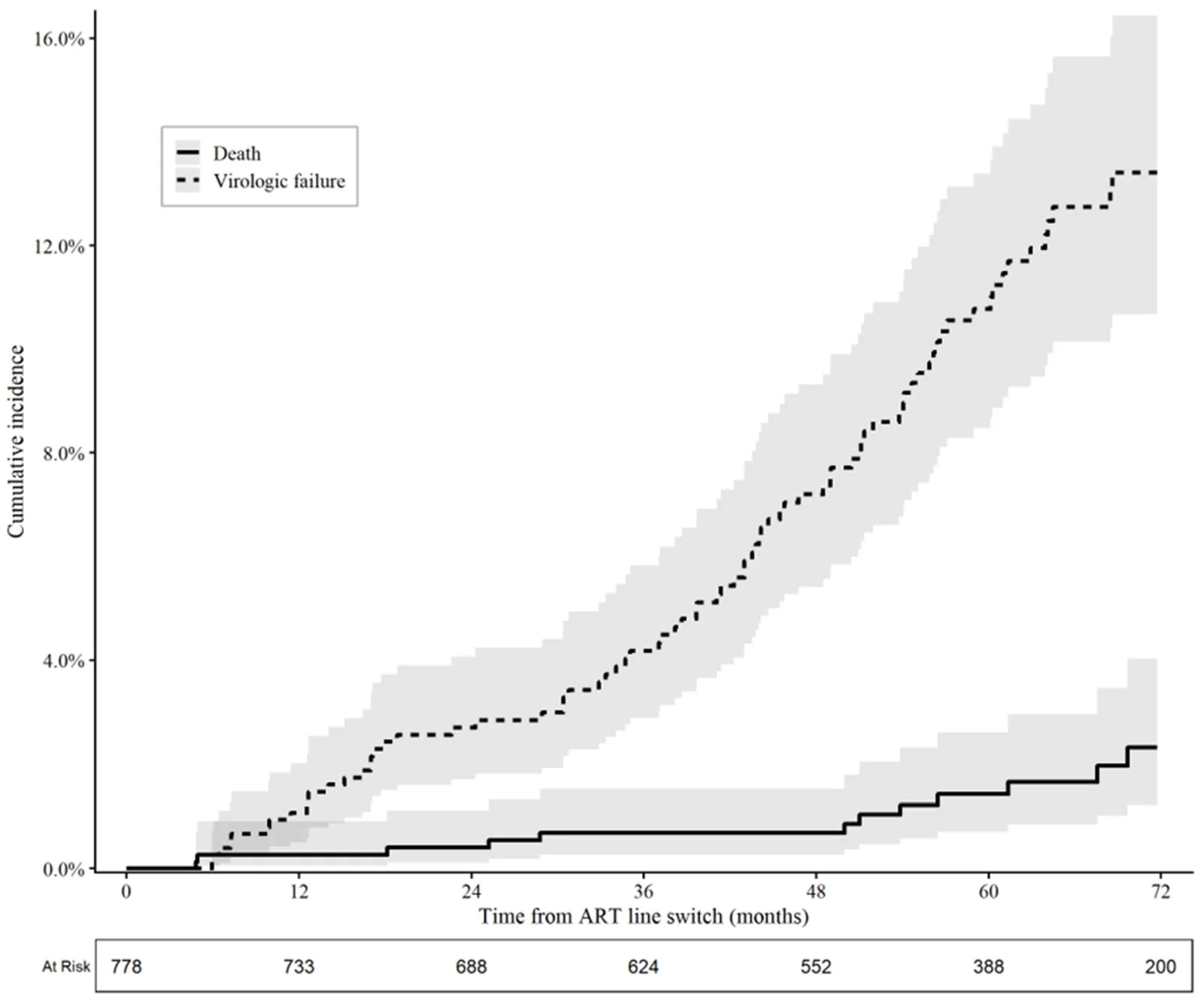
Cumulative incidence functions (Aalen–Johansen estimator) for virologic failure and all-cause death as competing events. Rwanda CBS cohort, 2019–2025. Shaded bands = 95% confidence intervals. Numbers at risk are displayed below the figure.

Event-free survival declined during follow-up (Figure 1A). Curves differed by regimen class, with lower event-free survival among patients receiving PI-based than INSTI-based regimens (log-rank *p* < 0.001; Figure 1B). Event-free survival also differed by CD4 category and WHO clinical stage at transition (both log-rank *p* < 0.001; Figure 2). Figure 3 presents cumulative incidence functions for VF and recorded death as first events.

### 3.4. Cox Proportional Hazards Regression

In the adjusted Cox model, three factors were independently associated with the composite outcome: PI-based regimen class (aHR 2.14, 95% CI 1.37–3.36, *p* < 0.001), WHO clinical stage III (aHR 1.92, 95% CI 1.10–3.34, *p* = 0.02), and WHO clinical stage IV (aHR 3.59, 95% CI 1.61–8.02, *p* = 0.002). Sex, age group, CD4 count category, marital status, and employment status were not independently associated with the composite outcome in the adjusted model. Unadjusted and adjusted hazard ratios are presented in Table 3.

### 3.5. Competing-Risks Analysis

The Fine–Gray model for VF, with death treated as a competing event, yielded a similar pattern to the composite-outcome Cox model. WHO stage IV was associated with a higher VF subdistribution hazard (sHR 4.44, 95% CI 1.91–10.4; *p* < 0.001), as was a PI-based regimen (sHR 3.37, 95% CI 1.96–5.78; *p* < 0.001). No death-specific Fine–Gray model was fitted because only 13 deaths occurred as first events (Table 4).

## 4. Discussion

In this national cohort of 778 PLWH transitioned to second- or third-line ART, 12.1% experienced the composite first event of the operational VF endpoint or recorded death over 3564 person-years. VF was the predominant first event, while recorded mortality was low. PI-based regimen use and advanced WHO clinical stage at transition were associated with higher hazards of the composite outcome, and PI-based regimen use and WHO stage IV were associated with a higher cumulative incidence of VF when death was treated as a competing event. These estimates describe associations rather than causal effects, particularly because regimen selection may have reflected prior treatment history, resistance, tolerability, or clinical severity.

The observed VF incidence was lower than estimates reported in several second-line ART cohorts and reviews from sub-Saharan Africa [8,9,21,22], although direct comparison is limited by differences in VF definitions, viral load testing schedules, follow-up, and patient selection. The higher risk among patients receiving PI-based regimens is directionally consistent with trial evidence showing strong virological performance of DTG-based second-line therapy, including DAWNING and NADIA [23,24]. However, those trials do not establish that all patients receiving PI-based therapy in routine care should switch to an INSTI; the present association may be partly explained by confounding by indication, unmeasured resistance, prior regimen failure, or adherence.

*Per national ART guidelines,* in this cohort, the PI-based regimen class consisted entirely of ritonavir-boosted ATV (*n* = 333, 42.8%) and ritonavir-boosted LPV (*n* = 17, 2.2%); ritonavir-boosted DRV (*n* = 21, 2.7%) was classified among the INSTI-based regimens, based on the anchor drug (RAL) (Table S4). The observed PI-based association therefore reflects an ATV/r-specific signal rather than a boosted-PI-class effect more broadly. The association between advanced WHO stage and adverse outcomes is consistent with evidence linking advanced HIV disease to treatment failure, opportunistic illness, and mortality [25,26,27,28]. The low recorded mortality rate may reflect Rwanda’s strong treatment programme, but it may also be influenced by survivor selection and incomplete ascertainment of deaths occurring after loss to follow-up [21,22,29,30,31,32].

For programmes and clinicians, the findings support closer viral load follow-up, structured adherence assessment, drug–drug interaction review, and access to resistance testing for patients receiving PI-based regimens, especially those with repeated non-suppression. Regimen changes should be individualized and resistance-informed rather than based on regimen class alone. Patients transitioning with WHO stage III or IV disease should receive the full advanced HIV disease package, including screening and treatment for opportunistic infections, prophylaxis where indicated, nutritional assessment, and intensified clinical follow-up [28]. At the programme level, CBS should capture the date and reason for regimen transition, baseline and repeat viral load results, adherence interventions, resistance results, transfer status, and verified mortality outcomes to support safer treatment decisions and more valid surveillance analyses.

This study’s strengths include its use of national longitudinal surveillance data, extended follow-up, and complementary Cox and competing-risks analyses. Important limitations nevertheless affect interpretation. The primary endpoint used one VL >1000 copies/mL and therefore represents an operational VF proxy rather than fully confirmed WHO-defined failure; the first 180 days contributed follow-up time although VF could not yet be ascertained. Adherence was missing for 84% of patients because CBS records this categorical assessment only when documented at the clinician’s discretion during a visit, without capturing the underlying counselling criteria such as pill count or missed-dose recall (Table S5). Viral load testing after transition was similarly inconsistent: approximately 15% of patients had no eligible test recorded in the 180-day-and-later window, and among those tested, the median time to the first eligible test exceeded the 6–12-month interval implied by national guidance (Table S6). Also, resistance, prior regimen history, tuberculosis, comorbidities, and facility-level clinical factors were similarly unavailable, leaving substantial potential for residual confounding and confounding by indication. Loss to follow-up may have concealed deaths or VF; extreme-value bounding showed that even a 10% unrecorded event rate among censored patients would nearly double the composite incidence rate, indicating that this remains an important source of uncertainty. The small numbers of deaths and third-line patients limited endpoint-specific analyses, and events-per-parameter was below the conventional threshold of 10 for both the composite (6.3) and VF-specific (5.4) models, indicating wider, less stable confidence intervals for lower-frequency categories. Under the sensitivity VF definition requiring two consecutive elevated viral loads (Table S3), the PI-based regimen association was directionally consistent but attenuated (aHR 1.72, 95% CI 0.98–3.04) compared with the primary single-VL definition, suggesting that some primary-endpoint events reflect transient viraemia (“blips”) rather than confirmed failure.

Future research should repeat the analysis using confirmed VF as the primary endpoint and a design that accounts explicitly for the six-month period before VF can be ascertained, such as a prespecified six-month landmark or delayed-entry approach. Linkage with laboratory, resistance-testing, pharmacy, tuberculosis, and civil-registration data would help distinguish biological failure from adherence-related non-suppression and improve mortality ascertainment. Regimen class and social variables should be measured at transition or modelled as time-varying exposures, and future analyses should address confounding by indication, facility-level clustering, informative loss to follow-up, and the growing third-line cohort.

## 5. Conclusions

Among PLWH transitioned to second- or third-line ART in Rwanda, PI-based regimen use—predominantly ATV/r—and advanced WHO clinical stage were associated with a higher risk of the composite outcome and the operational VF endpoint. Because regimen allocation was not randomized and resistance data were unavailable, the regimen association should inform intensified monitoring and resistance-guided clinical review rather than be interpreted as a causal comparison. The low recorded mortality rate should also be interpreted cautiously given the small number of events and possible under-ascertainment. Strengthening confirmed VF ascertainment, adherence and resistance data, and linkage to mortality sources will improve the value of CBS for guiding advanced ART care.

## Figures and Tables

**Table 1:** Baseline characteristics of PLWH transitioned to second- or third-line ART in Rwanda, 2019–2025 (*n* = 778).

Characteristic	*n* (%) or Median (IQR)	N
Age at ART line transition, years—median (IQR)	44 (33–51)	778
Age group, years		
<25	107 (14%)	
25–34	113 (15%)	
35–44	200 (26%)	
45–54	218 (28%)	
55+	140 (18%)	
Sex		
Female	519 (67%)	778
Male	259 (33%)	
Marital status		
Single	146 (19%)	778
In union	417 (54%)	
Separated/Divorced	69 (8.9%)	
Widowed	146 (19%)	
Employment status		
Employed	493 (63%)	778
Unemployed	285 (37%)	
WHO clinical stage		
I	517 (66%)	778
II	110 (14%)	
III	126 (16%)	
IV	25 (3.2%)	
CD4 count at transition, cells/µL		
<200	149 (19%)	778
200–499	340 (44%)	
≥500	262 (34%)	
Unknown	27 (3.5%)	
Median (IQR)	391 (247–591)	751
ART line at transition		
2nd line	757 (97%)	778
3rd line	21 (2.7%)	
ART regimen class after line transition		
INSTI-based	428 (55%)	778
PI-based	350 (45%)	
Worst recorded adherence during follow-up		
Good	76 (62%)	123
Moderate	25 (20%)	
Bad	22 (18%)	
Missing	655	

ART: antiretroviral therapy; INSTI: integrase strand transfer inhibitor; IQR: interquartile range; PI: protease inhibitor. Values are *n* (%) unless otherwise stated. N = number of patients with non-missing data. Marital and employment status reflect the most recent recorded value. Adherence recorded by attending clinician; 655 patients (84%) had no adherence record.

**Table 2:** Events, person-time, and crude incidence rates among PLWH transitioned to second- or third-line ART, Rwanda CBS, 2019–2025.

Outcome	N (%)	Person-Years	Incidence Rate per 100 PY (95% CI)
Virologic failure (VF) [VL > 1000 copies/mL]	81 (10.4%)	3185	2.54 (1.99–3.10)
All-cause death	13 (1.7%)	3564	0.36 (0.17–0.56)
Composite (VF or death)	94 (12.1%)	3564	2.64 (2.10–3.17)
Censored (alive, LTFU, or transfer-out)	684 (87.9%)	-	-

CBS: case-based surveillance; CI: confidence interval; LTFU: lost to follow-up; PY: person-years; VF: virological failure; VL: viral load. Median follow-up: 60.0 months. VF person-time counts only time after the 6-month (180 days) ascertainment window (delayed entry), since VF cannot be observed before then; death and the composite outcome use the total follow-up time from transition. The 13 deaths shown are those recorded as the FIRST event; 17 unique deaths occurred at any point during follow-up overall (the remainder occurred after a recorded VF event and are not double-counted here). Of the 684 censored, 679 remained in active care at last contact, and 5 were recorded lost to follow-up.

**Table 3:** Cox proportional hazards regression—predictors of virologic failure or death (composite outcome) among PLWH transitioned to second- or third-line ART, Rwanda CBS, 2019–2025.

Characteristic	N	HR (95% CI)	*p*-Value	aHR (95% CI)	*p*-Value
Sex					
Male (ref)	778	1.00		1.00	
Female		0.70 (0.46, 1.05)	0.08	0.72 (0.46, 1.14)	0.17
Age group, years					
<35 (ref)	778	1.00		1.00	
35–54		0.72 (0.46, 1.12)	0.15	0.90 (0.48, 1.66)	0.73
55+		0.59 (0.31, 1.12)	0.11	0.86 (0.40, 1.83)	0.70
WHO clinical stage					
I (ref)	778	1.00		1.00	
II		1.56 (0.88, 2.79)	0.13	1.66 (0.94, 2.89)	0.08
III		2.01 (1.21, 3.32)	**0.007**	1.92 (1.10, 3.34)	**0.02**
IV		3.94 (1.86, 8.32)	**<0.001**	3.59 (1.61, 8.02)	**0.002**
CD4 count at transition, cells/µL					
≥500 (ref)	778	1.00		1.00	
<200		0.98 (0.55, 1.75)	0.96	0.75 (0.41, 1.39)	0.37
2001.00499		0.90 (0.57, 1.43)	0.65	0.85 (0.53, 1.35)	0.49
Unknown		1.08 (0.33, 3.51)	0.90	0.97 (0.29, 3.19)	0.94
ART regimen class after line transition					
INSTI-based (ref)	778	1.00		1.00	
PI-based		2.25 (1.47, 3.45)	**<0.001**	2.14 (1.37, 3.36)	**<0.001**
Marital status					
Single (ref)	778	1.00		1.00	
In union		0.73 (0.45, 1.18)	0.20	0.88 (0.46, 1.68)	0.70
Separated/Divorced		0.85 (0.41, 1.78)	0.66	0.90 (0.35, 2.31)	0.82
Widowed		0.34 (0.16, 0.74)	**0.006**	0.51 (0.20, 1.27)	0.15
Employment status					
Employed (ref)	778	1.00		1.00	
Unemployed		1.04 (0.68, 1.57)	0.87	0.97 (0.65, 1.44)	0.86

aHR: adjusted hazard ratio; CI: confidence interval; HR: hazard ratio; INSTI: integrase strand transfer inhibitor; PI: protease inhibitor; ref: reference category. Bold *p*-values indicate *p* < 0.05. Adjusted model includes sex, age group, WHO clinical stage, CD4 count, ART regimen class at transition, marital status, and employment status; robust standard errors clustered by health facility. Adjusted model events-per-parameter (EPV) = 6.3 (94 events/15 parameters); EPV below conventional threshold of ~10 indicates that wider, less stable confidence intervals should be expected for some categories.

**Table 4:** Fine–Gray subdistribution hazard ratios for virologic failure (death as competing event) among PLWH transitioned to second- or third-line ART, Rwanda CBS, 2019–2025.

Characteristic	sHR (95% CI)	*p*-Value
Sex		
Male (ref)	1.00	
Female	0.87 (0.52, 1.45)	0.60
Age group, years		
<35 (ref)	1.00	
35–54	0.97 (0.51, 1.87)	0.94
55+	0.76 (0.31, 1.83)	0.53
WHO clinical stage		
I (ref)	1.00	
II	1.46 (0.73, 2.92)	0.29
III	1.79 (0.98, 3.26)	0.06
IV	4.44 (1.91, 10.4)	**<0.001**
CD4 count at transition, cells/µL		
≥500 (ref)	1.00	
<200	0.69 (0.36, 1.34)	0.28
200–499	0.82 (0.48, 1.39)	0.46
Unknown	1.01 (0.31, 3.31)	>0.99
ART regimen class after line transition		
INSTI-based (ref)	1.00	
PI-based	3.37 (1.96, 5.78)	**<0.001**
Marital status		
Single (ref)	1.00	
In union	0.95 (0.47, 1.91)	0.88
Separated/Divorced	0.84 (0.30, 2.30)	0.73
Widowed	0.41 (0.14, 1.16)	0.09
Employment status		
Employed (ref)	1.00	
Unemployed	1.01 (0.65, 1.53)	0.98

sHR: subdistribution hazard ratio; CI: confidence interval; INSTI: integrase strand transfer inhibitor; PI: protease inhibitor; ref: reference category. Bold *p*-values indicate *p* < 0.05. The model used delayed entry at 6 months (180 days) post-transition (the earliest VF can be ascertained); robust standard errors were clustered by health facility. Adjusted for sex, age group, WHO clinical stage, CD4 count, ART regimen class at transition, marital status, and employment status. Events-per-parameter (EPV) = 5.4 (81 VF events/15 parameters), on the low side of the conventional ~10 threshold. A Fine–Gray model for all-cause death was not fitted given the small number of death events (*n* = 13).

## Data Availability

The original contributions presented in this study are included in the article/Supplementary Materials. Further inquiries can be directed to the corresponding author.

## Supplementary Materials

The following supporting information can be downloaded at https://www.mdpi.com/article/10.3390/v18080905/s1, Figure S1: Kaplan-Meier event-free survival curve for the composite outcome under the sensitivity virologic failure definition (two consecutive VLs > 1000 copies/mL), Rwanda CBS cohort, 2019–2025; Table S1: Event counts and incidence rates under the sensitivity VF definition (two consecutive VLs > 1000 copies/mL), Rwanda CBS cohort, 2019–2025 (N = 778); Table S2: Extreme-value bounding analysis for informative censoring; Table S3: Adjusted Cox proportional hazards regression under the sensitivity VF definition (two consecutive VLs > 1000 copies/mL) among PLWH transitioned to second- or third-line ART, Rwanda CBS, 2019–2025; Table S4: Specific regimen (drug combination, including NRTI backbone) switched to at transition, among the full analytic cohort (N = 778); Table S5: Baseline characteristics and outcomes by availability of a recorded adherence value; Table S6: Viral load monitoring frequency and timing after ART line transition.

## References

[1] UNAIDS (2025). AIDS, Crisis and the Power to Transform: Global AIDS Update 2025.

[2] Assefa Y., Ooms G., Komatsu R., Woldeyohannes S., Gilks C.F. (2025). The successful scaling-up of antiretroviral therapy globally has many lessons for advancing universal health coverage: Progress at risk. Glob. Health.

[3] World Health Organization (2021). Consolidated Guidelines on HIV Prevention, Testing, Treatment, Service Delivery and Monitoring: Recommendations for a Public Health Approach.

[4] Namaganda M.M., Kafeero H.M., Nabende J.N., Kateete D.P., Batte C., Wanyengera M., Jjingo D., Joloba M., Kivunike F., Ssewanyana I. (2025). Prevalence and predictors of virological failure among the people living with HIV on antiretroviral treatment in East Africa: Evidence from a systematic review with meta-analysis and meta-regression of published studies from 2016 to 2023. HIV Res. Clin. Pract..

[5] Kiweewa F., Esber A., Musingye E., Reed D., Crowell T.A., Cham F., Semwogerere M., Namagembe R., Nambuya A., Kafeero C. (2019). HIV virologic failure and its predictors among HIV-infected adults on antiretroviral therapy in the African Cohort Study. PLoS ONE.

[6] Ayana W.G., Ayana Hordofa M., Dechasa Yadeta A. (2024). Determinants of virologic failure among adult HIV patients on first line antiretroviral treatment in Oromia, Central Ethiopia: 2022 a case-control study. AIDS Res. Ther..

[7] Zerihun E., Tesema K., Abera F. (2025). Virological treatment failure and associated factors among adults on first-line antiretroviral therapy in West Hararghe, Ethiopia. Front. Public Health.

[8] Mwavika E.T., Kunambi P.P., Masasi S.J., Lema N., Kamori D., Matee M. (2024). Prevalence, rate, and predictors of virologic failure among adult HIV-Infected clients on second-line antiretroviral therapy (ART) in Tanzania (2018–2020): A retrospective cohort study. Bull. Natl. Res. Cent..

[9] Edessa D., Sisay M., Asefa F. (2019). Second-line HIV treatment failure in sub-Saharan Africa: A systematic review and meta-analysis. PLoS ONE.

[10] Venter W.D.F., Sokhela S., Simmons B., Moorhouse M., Fairlie L., Mashabane N., Serenata C., Akpomiemie G., Masenya M., Qavi A. (2020). Dolutegravir with emtricitabine and tenofovir alafenamide or tenofovir disoproxil fumarate versus efavirenz, emtricitabine, and tenofovir disoproxil fumarate for initial treatment of HIV-1 infection (ADVANCE): Week 96 results from a randomised, phase 3, non-inferiority trial. Lancet HIV.

[11] Osiyemi O., De Wit S., Ajana F., Bisshop F., Portilla J., Routy J.P., Wyen C., Ait-Khaled M., Leone P., Pappa K.A. (2022). Efficacy and Safety of Switching to Dolutegravir/Lamivudine Versus Continuing a Tenofovir Alafenamide–Based 3- or 4-Drug Regimen for Maintenance of Virologic Suppression in Adults Living with Human Immunodeficiency Virus Type 1: Results Through Week 144 From the Phase 3, Noninferiority TANGO Randomized Trial. Clin. Infect. Dis..

[12] Lockman S., Brummel S.S., Ziemba L., Stranix-Chibanda L., McCarthy K., Coletti A., Jean-Philippe P., Johnston B., Krotje C., Fairlie L. (2021). Efficacy and safety of dolutegravir with emtricitabine and tenofovir alafenamide fumarate or tenofovir disoproxil fumarate, and efavirenz, emtricitabine, and tenofovir disoproxil fumarate HIV antiretroviral therapy regimens started in pregnancy (IMPAACT 2010/VESTED): A multicentre, open-label, randomised, controlled, phase 3 trial. Lancet.

[13] Cahn P., Madero J.S., Arribas J.R., Antinori A., Ortiz R., Clarke A.E., Hung C.-C., Rockstroh J.K., Girard P.-M., Sievers J. (2019). Dolutegravir plus lamivudine versus dolutegravir plus tenofovir disoproxil fumarate and emtricitabine in antiretroviral-naive adults with HIV-1 infection (GEMINI-1 and GEMINI-2): Week 48 results from two multicentre, double-blind, randomised, non-inferiority, phase 3 trials. Lancet.

[14] de Waal R., Lessells R., Hauser A., Kouyos R., Davies M.-A., Egger M., Wandeler G. (2018). HIV drug resistance in sub-Saharan Africa: Public health questions and the potential role of real-world data and mathematical modelling. J. Virus Erad..

[15] Oluoch T., Byiringiro B., Tuyishime E., Kitema F., Ntwali L., Malamba S., Wilmore S., Remera E. (2024). Implementation of an HIV Case Based Surveillance Using Standards-Based Health Information Exchange in Rwanda. Stud. Health Technol. Inf..

[16] Rwanda Ministry of Health and RBC National HIV, STIs and Viral Hepatitis Annual Report 2023–2024. https://rbc.gov.rw/fileadmin/user_upload/report_2026/HIV%20Annual%20report%202023-2024L.pdf.

[17] Ayenew G., Agumas Y., Shibabaw T., Getaneh G., Getie M. (2024). Determinants of virological failure among HIV clients on second-line antiretroviral treatment at Felege-hiwot and University of Gondar comprehensive specialized hospitals in the Amhara Region, Northwest Ethiopia: A case-control study. PLoS ONE.

[18] Abuhay H.W., Endalew T., Birhan T.Y., Muche A.A. (2024). Time to Treatment Failure and Its Predictors Among Second-Line ART Clients in Amhara Region, Ethiopia: A Retrospective Follow-Up Study. HIV/AIDS.

[19] Fine J.P., Gray R.J. (1999). A Proportional Hazards Model for the Subdistribution of a Competing Risk. J. Am. Stat. Assoc..

[20] National Institute of Statistics of Rwanda (NISR), National Institute of Statistics of Rwanda, Rwanda, DHS Program (2022). The Fifth Rwanda Population and Housing Census, Main Indicators Report, February 2023. National Institute of Statistics of Rwanda, Ministry of Finance and Economic Planning: Ministry of Health.

[21] Tsegaye A.T., Alemu W., Ayele T.A. (2019). Incidence and determinants of mortality among adult HIV infected patients on second-line antiretroviral treatment in Amhara region, Ethiopia: A retrospective follow up study. Pan Afr. Med. J..

[22] Kidie A.A., Masresha S.A., Bizuneh F.K. (2024). Statistical analysis on the incidence and predictors of death among second-line ART patients in public hospitals of North Wollo and Waghemira Zones, Ethiopia, 2021. Sci. Rep..

[23] Paton N.I., Musaazi J., Kityo C., Walimbwa S., Hoppe A., Balyegisawa A., Asienzo J., Kaimal A., Mirembe G., Lugemwa A. (2022). Efficacy and safety of dolutegravir or darunavir in combination with lamivudine plus either zidovudine or tenofovir for second-line treatment of HIV infection (NADIA): Week 96 results from a prospective, multicentre, open-label, factorial, randomised, non-inferiority trial. Lancet HIV.

[24] Aboud M., Kaplan R., Lombaard J., Zhang F., Hidalgo J.A., Mamedova E., Losso M.H., Chetchotisakd P., Brites C., Sievers J. (2019). Dolutegravir versus ritonavir-boosted lopinavir both with dual nucleoside reverse transcriptase inhibitor therapy in adults with HIV-1 infection in whom first-line therapy has failed (DAWNING): An open-label, non-inferiority, phase 3b trial. Lancet Infect. Dis..

[25] Aytenew T.M., Asferie W.N., Ejigu N., Birhane B.M., Tiruneh Y.M., Kassaw A., Asnakew S., Legas G., Munie B.M., Belay B.M. (2024). Virological failure and associated factors among patients receiving anti-retroviral therapy in Ethiopia: A systematic review and meta-analysis. BMJ Open.

[26] Abebe A.D., Tassew W.C., Ferede Y.A., Beyene J.A., Assefa M. (2025). Prevalence and determinants of virologic failure among HIV patients on ART in Africa: A systematic review and meta-analysis. Virol. J..

[27] Ford N., Kassanjee R., Stelzle D., Jarvis J.N., Sued O., Perrin G., Doherty M., Rangaraj A. (2025). Global prevalence of advanced HIV disease in healthcare settings: A rapid review. J. Int. AIDS Soc..

[28] World Health Organization (2025). WHO Guidelines on the Management of Advanced HIV Disease.

[29] Nsanzimana S., Remera E., Kanters S., Chan K., Forrest J.I., Ford N., Condo J., Binagwaho A., Mills E.J. (2015). Life expectancy among HIV-positive patients in Rwanda: A retrospective observational cohort study. Lancet Glob. Health.

[30] Inzaule S.C., Kroeze S., Kityo C.M., Siwale M., Akanmu S., Wellington M., de Jager M., Ive P., Mandaliya K., Stevens W. (2022). Long-term HIV treatment outcomes and associated factors in sub-Saharan Africa: Multicountry longitudinal cohort analysis. AIDS.

[31] Scheier T.C., De Gouveia K., Engel M.E., Hohlfeld A.S.-J., Cen A., Berhe A., Fan S., Li J., Elliott S., Ford N. (2026). One-year mortality among adults with advanced HIV in sub-Saharan Africa: A systematic review and meta-analysis. AIDS.

[32] Ballif M., Christ B., Anderegg N., Chammartin F., Muhairwe J., Jefferys L., Hector J., van Dijk J., Vinikoor M.J., van Lettow M. (2022). Tracing People Living with Human Immunodeficiency Virus Who Are Lost to Follow-up at Antiretroviral Therapy Programs in Southern Africa: A Sampling-Based Cohort Study in 6 Countries. Clin. Infect. Dis. Off. Publ. Infect. Dis. Soc. Am..

