# Supplementary material for: Virological Failure and Mortality Among People Living with HIV Transitioned to Second- or Third-Line Antiretroviral Therapy in Rwanda: A Competing-Risks Survival Analysis of National Case-Based Surveillance Data, 2019–2025
Source: Viruses. 2026 Aug 17;18(8):905. doi: 10.3390/v18080905 (PMC13517693; doi:10.3390/v18080905)
Supplement: Supplementary file 1 [file viruses-18-00905-s001.zip › viruses-4439489-supplementary.pdf]

Supplementary tables

Table S1. Event counts and incidence rates under the sensitivity VF definition (two consecutive VLs > 1,000 copies/mL), Rwanda CBS cohort, 2019-2025 (N = 778).

Outcome	N (%)	Incidence rate per 100 PY (95% CI)
Confirmed VF (2 consecutive VL > 1,000 copies/mL)	44 (5.7%)	1.22 (0.86-1.58)
All-cause death	14 (1.8%)	0.39 (0.18-0.59)
**Composite (confirmed VF or death)**	**58 (7.5%)**	**1.60 (1.19-2.02)**

CI: confidence interval; PY: person-years; VF: virologic failure; VL: viral load. Median follow-up: 61.4 months.

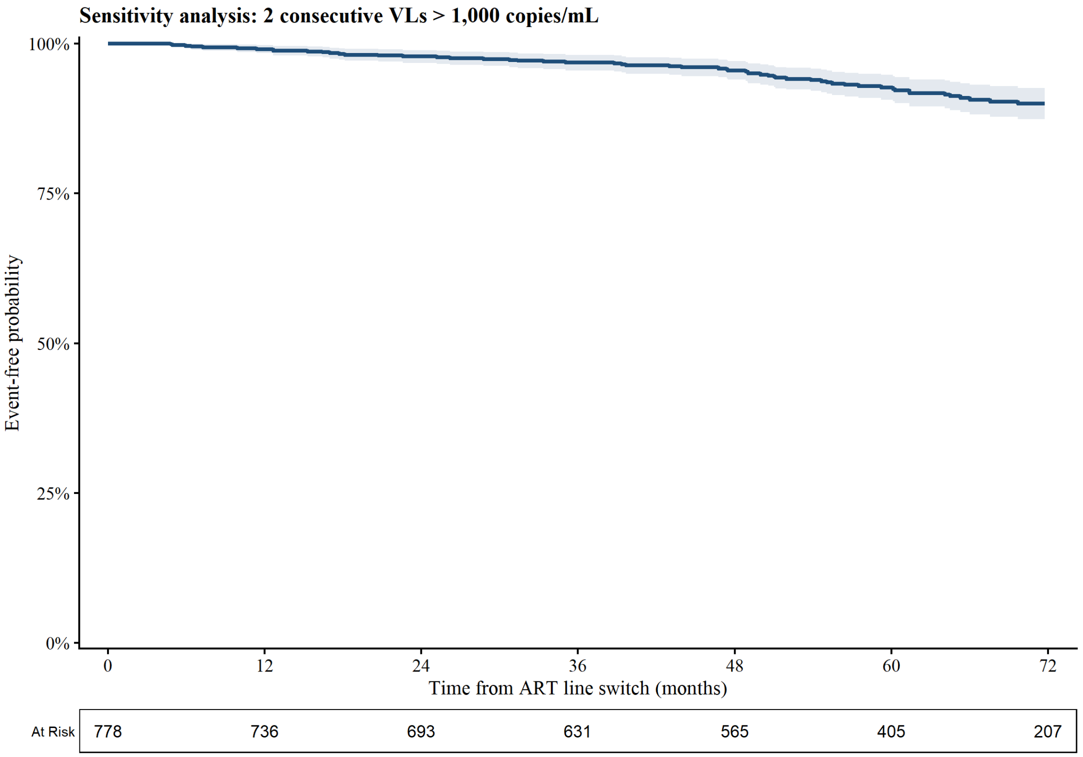

Figure S1. Kaplan-Meier event-free survival curve for the composite outcome under the sensitivity virologic failure definition (two consecutive VLs > 1,000 copies/mL), Rwanda CBS cohort, 201s9-2025. *Shaded band = 95% confidence interval. Numbers at risk displayed below the figure.*

Table S2. Extreme-value bounding analysis for informative censoring.

Scenario	N reclassified	Composite events (N)	Incidence rate per 100 PY (95% CI)
0% of censored patients assumed to have an unrecorded event	0	94	2.64 (2.10–3.17)
10% of censored patients assumed to have an unrecorded event	68	162	4.54 (3.85–5.24)

25% of censored patients assumed to have an unrecorded event	171	265	7.43 (6.54–8.33)

PY: person-years. composite-outcome incidence rate recomputed under illustrative scenarios where a proportion of censored patients are assumed to have experienced an unrecorded VF or death event at their last observed contact. The assumption that 10% of censored patients experienced an unrecorded event nearly doubles the estimated composite incidence rate.

Table S3. Adjusted Cox proportional hazards regression under the sensitivity VF definition (two consecutive VLs > 1,000 copies/mL) among PLWH transitioned to second- or third-line ART, Rwanda CBS, 2019-2025.

Characteristic	aHR (95% CI)	p-value
**Sex**		
Male (ref)	1.00	
Female	0.67 (0.38, 1.18)	0.17
**Age group, years**		
<35 (ref)	1.00	
35–54	0.90 (0.42, 1.96)	0.80
55+	1.11 (0.46, 2.68)	0.82
**WHO clinical stage**		
I (ref)	1.00	
II	1.92 (1.04, 3.57)	**0.04**
III	1.77 (0.89, 3.49)	0.10
IV	1.90 (0.54, 6.73)	0.32
**CD4 count at transition, cells/μL**		
≥500 (ref)	1.00	
<200	1.32 (0.58, 2.96)	0.51
200-499	1.16 (0.58, 2.33)	0.67
Unknown	1.35 (0.38, 5.48)	0.68
**ART regimen class after line transition**		
INSTI-based (ref)	1.00	
PI-based	1.65 (0.93, 2.91)	0.09
**Marital status (most recent)**		
Single (ref)	1.00	
In union	0.62 (0.27, 1.41)	0.25
Separated/Divorced	0.81 (0.26, 2.51)	0.71
Widowed	0.39 (0.13, 1.19)	0.10
**Employment status (most recent)**		
Employed (ref)	1.00	

Unemployed	1.08 (0.62, 1.87)	0.79

aHR: adjusted hazard ratio; CI: confidence interval; INSTI: integrase strand transfer inhibitor; PI: protease inhibitor; ref: reference category. Bold p-values indicate p<0.05. Model adjusted for the same covariates as the primary model; sensitivity-definition events were too sparse in this subgroup to estimate a stable coefficient (quasi-complete separation).

Table S4. Specific regimen (drug combination, including NRTI backbone) switched to at transition, among the full analytic cohort (N=778).

Regimen (as switched to)	Frequency n(%)
AZT+3TC+ATV/r	333 (42.8%)
AZT+3TC+DTG	407 (52.3%)
AZT+3TC+LPV/r	17 (2.2%)
RAL+DRV/r+TDF	8 (1.0%)
RAL+ETV+DRV/r	13 (1.7%)

NRTI: 3TC = lamivudine; AZT = zidovudine; TDF = tenofovir disoproxil fumarate; INSTI: DTG = dolutegravir; RAL = raltegravir; PI: ATV/r ('/r' = ritonavir-boosted) = atazanavir; DRV/r = darunavir/ritonavir; LPV/r = lopinavir/ritonavir ('/r' = ritonavir-boosted); NNRTI: ETV = etravirine. ATV and LPV are ritonavir-boosted at 100 mg once daily as part of second-line ART, while DRV is ritonavir-boosted at 100 mg twice daily as part of third-line ART. RAL+DRV/r+TDF and RAL+ETV+DRV/r were classified as INSTI-based regimens.

Table S5. Baseline characteristics and outcomes by availability of a recorded adherence value

Characteristic	Recorded N = 123	Missing N = 655	p-value[1]
**Age at ART line switch, years — median (IQR)**	40 (29–48)	44 (34–52)	**0.007**
**Age group, years, n (%)**			0.19
<25	22 (18%)	85 (13%)	
25–34	24 (20%)	89 (14%)	
35–44	29 (24%)	171 (26%)	
45–54	30 (24%)	188 (29%)	
55+	18 (15%)	122 (19%)	
**Sex, n (%)**			0.06
Female	91 (74%)	428 (65%)	
Male	32 (26%)	227 (35%)	
**Marital status (nearest to transition), n (%)**			**0.04**
Single	32 (26%)	114 (17%)	
In union	57 (46%)	360 (55%)	
Separated/Divorced	15 (12%)	54 (8.2%)	
Widowed	19 (15%)	127 (19%)	

Characteristic	Recorded<br>N = 123	Missing<br>N = 655	p-value[1]
**Employment status (nearest to transition), n (%)**			0.67
Employed	80 (65%)	413 (63%)	
Unemployed	43 (35%)	242 (37%)	
**WHO clinical stage, n (%)**			0.23
I	80 (65%)	437 (67%)	
II	20 (16%)	90 (14%)	
III	16 (13%)	110 (17%)	
IV	7 (5.7%)	18 (2.7%)	
**CD4 count at enrolment, cells/µL, n (%)**			**0.01**
<200	28 (23%)	121 (18%)	
200–499	44 (36%)	296 (45%)	
≥500	41 (33%)	221 (34%)	
Unknown	10 (8.1%)	17 (2.6%)	
**ART line at switch, n (%)**			0.36
2nd line	118 (96%)	639 (98%)	
3rd line	5 (4.1%)	16 (2.4%)	
**ART regimen class at transition, n (%)**			**0.04**
INSTI-based	57 (46%)	371 (57%)	
PI-based	66 (52%)	284 (41%)	
**Virologic failure, n (%)**	21 (17%)	60 (9.2%)	**0.008**
**Death (first event), n (%)**	3 (2.4%)	10 (1.5%)	0.44
**Composite outcome (VF or death), n (%)**	24 (20%)	70 (11%)	**0.006**

[1]Wilcoxon rank sum test; Pearson's Chi-squared test; Fisher's exact test. Adherence was missing for 655 of 778 patients (84.2%). P values test for a difference between patients with and without a recorded adherence value.

Table S6. Viral load monitoring frequency and timing after ART line transition.

Metric	Value
Patients with ≥1 eligible VL test after the 180-day window	661 / 778 (85.0%)
Number of eligible VL tests per patient, median (IQR) — among those tested	4 (2–7)
Days from transition to first eligible VL test, median (IQR)	1167 (619–1473)

VL: viral load. Approximately 15% of patients had no eligible VL test recorded at or after the 180-day post-transition window; among those tested, the median time to first eligible test exceeded the 6–12 month interval implied by national guidance, consistent with real-world monitoring gaps. Per national ART guidelines, monitoring intervals are protocol-defined and do not vary by regimen class; results are therefore reported overall rather than stratified by regimen.
